# Enhancing 5G propagation into vehicles and buildings using optically transparent and polarisation insensitive metasurfaces over wide-incidence angles

**DOI:** 10.1038/s41598-024-51447-3

**Published:** 2024-03-21

**Authors:** Amirmasood Bagheri, Shadi Danesh, Fan Wang, Seyed Ehsan Hosseininejad, Mohsen Khalily, Rahim Tafazolli

**Affiliations:** 1 5G & 6G Innovation Centres (5GIC & 6GIC), Institute for Communication Systems (ICS), University of Surrey, Guildford, GU2 7XH UK; 2 Shanghai Huawei Technologies Company Ltd, Shanghai, 201206 China

**Keywords:** Electrical and electronic engineering, Engineering

## Abstract

This article introduces two transmissive metasurfaces applied to normal windows, aiming to improve the 5G outdoor-to-indoor (O2I) coverage. These windows can be utilized in various settings, such as vehicles or buildings. The proposed unit cells, designed to be wide-incident angle and polarization insensitive, are implemented in both single-glazing and double-glazing glasses, arranged in a periodic structure to form the transmission surfaces. Both metasurfaces maintain optical transparency by incorporating Indium Tin Oxide (ITO) as the conductive element in each unit cell. These engineered transmission surfaces enhance the 5G signal indoor coverage at the 3.5 GHz band across a broad range of incident angles. While multi-layer structures typically exhibit heightened sensitivity to the angle of incidence, the proposed two-layered transmissive surfaces demonstrate substantial angular stability, reaching up to 65 and 75 degrees for double- and single-glazed glass, respectively. To achieve this wide and stable angular response, evolutionary optimization techniques were employed to fine-tune the proposed unit cells. Both designs exhibit a high transmission coefficient across the operating frequency for a variety of incident angles, surpassing those reported in the existing literature. Experimental evaluations of the fabricated prototypes indicate that both metasurfaces hold significant potential for enhancing signal propagation into buildings and vehicles.

## Introduction

The Fifth-generation (5G) network and 5G wireless devices are advancing at an inconceivable rate today. 5G cellular networks capitalize on the extensive bandwidth available in the sub-6 GHz band. The evolution of 5G holds the potential for sophisticated, hyper-scale haptic Internet-of-Things (IoT) technologies featuring low latency and high data rates. Additionally, it introduces new forms of connectivity, such as outdoor-to-indoor (O2I), communication between vehicles and infrastructure (V2I), vehicles and vehicles (V2V), and interaction between pedestrians and individuals. In the initial phase of 5G development, priority was given to indoor locations as crucial coverage areas. This emphasis on indoor areas persists from the 4G era, recognizing them as significant traffic hotspots^1^. A recent report reveals that 90% of the time, people spend indoors and 80% of mobile Internet access traffic occurs indoors^2^. Consequently, one of the main missions of 5G is to increase capacity and enhance the user experience in indoor environments. Achieving seamless wireless indoor coverage necessitates ensuring adequate signal strength. However, numerous challenges confront service providers in delivering wireless coverage, especially to a large number of indoor users. User Equipment (UE) transmit power restrictions further compound the coverage limitations. Essentially, the loss from the User Equipment’s perspective inside the building is attributed to reflected waves by the outer interface of the wall. Moreover, substantial loss occurs due to the shielding effect of building walls, resulting in high penetration losses that significantly degrade data rates, energy efficiency, and spectral efficiency. This considerable loss varies based on the dielectric properties of the building material, the incident angle of the radio wave to the surface material, polarization of the wave, and the operating frequency. The frequency range of 3.3 GHz to 3.8 GHz, or a portion thereof, is allocated for 5G use in various countries. Penetration loss through building materials, based on experimental data obtained from diverse structures at 3.5 GHz, indicates that the transmission coefficient for normal wave incidence on various concrete walls falls within the range of 25 to 35 dB. Similarly, conducting the same measurement on drywall yields a power transmission coefficient in the range of 10 to 15 dB, while a brick wall exhibits a transmission coefficient of 12 to 20 dB. These coefficients surpass the penetration losses observed through glass^3^. In the upcoming sections, the transmission coefficient from glass will be discussed. Given that glass has a lower transmission coefficient compared to other commonly used materials in typical buildings, adjusting the frequency response of this section of the building is viewed as a promising method to enhance indoor coverage. Additionally, glass can be assumed as a homogeneous material since its electrical properties are almost same at any two points in the material separated by distances even much less than any wavelength of interest in n77 and n78 bands. As an instance, wood, concrete, and brick all have some degrees of granularity, due either to an intrinsic mix of materials, or variations in the density or porosity of the material. Based on this concept, coating a window with optically transparent electromagnetic engineering transmission surfaces can be a flexible, simple and inexpensive solution to fully realize the potentials of 5G promising indoor network infrastructure. It’s important to emphasize that addressing the coverage challenge requires a solution that can be easily applied to existing windows, ensuring a straightforward installation process. Furthermore, the transmission coefficient is influenced by the incident angle of the radio wave, typically resulting in reduced transmission as the angle increases. The proposed structure, intended for coating on both single- and double-glazed glasses, must effectively mitigate angle and polarization dependencies, ensuring a consistently high transmission coefficient across the entire operating frequency band. Moreover, a preference is given to a fully passive design. While tunable designs offer flexibility, a completely passive approach is favored for its simplicity, making it more suitable for widespread use by normal users as opposed to being primarily tailored for service providers^4^.

Frequency selective surfaces (FSS), typically composed of periodic arrays of well-designed metallic apertures or patches, are commonly organized in single or multilayer configurations^5,6^. Owing to their thin profile, low cost, and simple designing procedure, they have been utilized in diverse applications, including filters, absorbers, antennas, radomes, polarizers^7–11^, electromagnetic (EM) compatibility, and interference cancellation^12,13^. However, their performance strictly depends on the polarization and incident angles of the EM wave, primarily due to the substantial dimensions of the resonant elements. Moreover, in practical applications, there is a preference for miniaturized FSS, where a significant number of unit cells must be accommodated within confined spaces to simulate the behavior of an infinite FSS^14,15^. Traditional FSSs are narrow band and they do not provide adequate spatial filtering response.

Hence, there has been considerable research focus on developing frequency selective surfaces (FSS) that are insensitive to polarization and incident angles, while also incorporating miniaturization features. Additionally, ongoing extensive research aims to further shrink the FSS profile while enhancing frequency response, achieving broader bandwidth even at higher incidence angles, capable of supporting, filtering, or converting various electromagnetic wave polarizations^16–20^. It must be noted that while single-layered Frequency selective surfaces (FSSs) are straightforward to fabricate and implement, they exhibit inefficiencies in terms of both amplitude and phase responses across frequencies^21^. In order to overcome the limitations of conventional single layered FSSs, multilayered FSSs have been introduced, which offer additional flexibility of varying parameters to obtain the desired performance^22,23^. Metasurface-based FSSs not only offer the advantage of accommodating a large number of unit cells within confined spaces, but also support the smoother frequency response in respect to incident angle. They are highly efficient for transmission surface printed on small apertures like a window to enhance the 5G indoor coverage. Recently, reflective and transmissive metasurfaces have been proposed and widely studied to ensure the seamless connectivity in both outdoor and indoor environment. The reflective one^4^ has been proposed to improve the outdoor coverage while the transmissive type has been suggested to improve the outdoor to indoor coverage. In Ref.^17^ a solution for O2I coverage enhancement is proposed in which small unit-cells $\bigg (\dfrac{\lambda }{30}< unit-cell_{size} < \dfrac{\lambda }{3}\bigg )$^24^, have been utilised. In this definition $\lambda$ is the free space wavelength at the central frequency of operation. Additionally, using this criteria, our final proposed structures can be named metasurface^24^. However, the proposed structure is limited only to single-glaze windows and also it can support up to only 50 degrees of incident angle and the transparency of structure is low ($\approx 58.84 \%$). In Ref.^18^ an FSS structure boasts a transparency of approximately ($\approx 76\%$), accommodating incident angles of up to $30^\circ$. However, it is limited to narrow single glazing windows. Despite this limitation, both designs share the desirable characteristic of polarization independence, a crucial feature for enhancing 5G outdoor-to-indoor (O2I) coverage.

In view of above aspects, polarization insensitive FSS is proposed in Ref.^25^ to provide incident wave angular stability up to 45^∘^. In addition, the design proposed in Ref.^26^ shows stable response up to 45^∘^. Although it transmits frequencies between 5 and 6 GHz and around 2.5 GHz, it completely blocks n77 and n78 frequency bands. The miniaturized FSS-based radome was reported in Ref.^27^ to reduce out-of-band RCS of antenna-radome system, but it exhibited narrow bandpass characteristics. Such type of FSS structures have been employed to achieve a wide bandwidth, although they are constrained to a maximum incidence angle of 50^∘^^28^.

In this paper, two wide-band, polarisation insensitive unit cells with broad incident angle capabilities have been proposed. These unit-cells are arranged in a 2D periodic structure and coated on both single and double-glaze windows. Despite the typically increased sensitivity of multilayer structures to the angle of incidence, our proposed two-layered metasurface exhibits improved angular stability, reaching up to 65 and 75 degrees for double and single glazing cases, respectively. This surpasses the performance of structures presented in the literature review.

Next, we will delve into the issue of O2I coverage and provide a corresponding discussion on the attenuation of signals through glass in both vehicles and buildings. Furthermore, a comprehensive optimization procedure utilizing the circuit model for Extraordinary Transmission (EOT) structures is outlined and applied to design appropriate unit cells. Later, we will discuss the proposed unit-cells and present the simulation and measured results for both developed metasurfaces.

## Enhancing propagation into vehicles and buildings

In this section, use of metasurface to enhance propagation through outside vehicles and walls is described. Propagation through glass has been discussed and investigated for single- and double-galzed glasses. This focus on glass is due to its lower attenuation compared to the brick and block constructions of buildings and the metallic body of any vehicle.

Generally commercial glasses can be divided into single, double and triple glazing. In this section based on the nominal dielectric constant of glasses which are also verified experimentally, transmission coefficient for different type of glasses are studied with numerical simulations in CST software. In our simulation dielectric constant of 6^29^ has been considered for both single- and double glazing glasses. The geometrical parameters are also extracted from commercially available glasses.

### Propagation through single glazing glass

**Figure 1:**
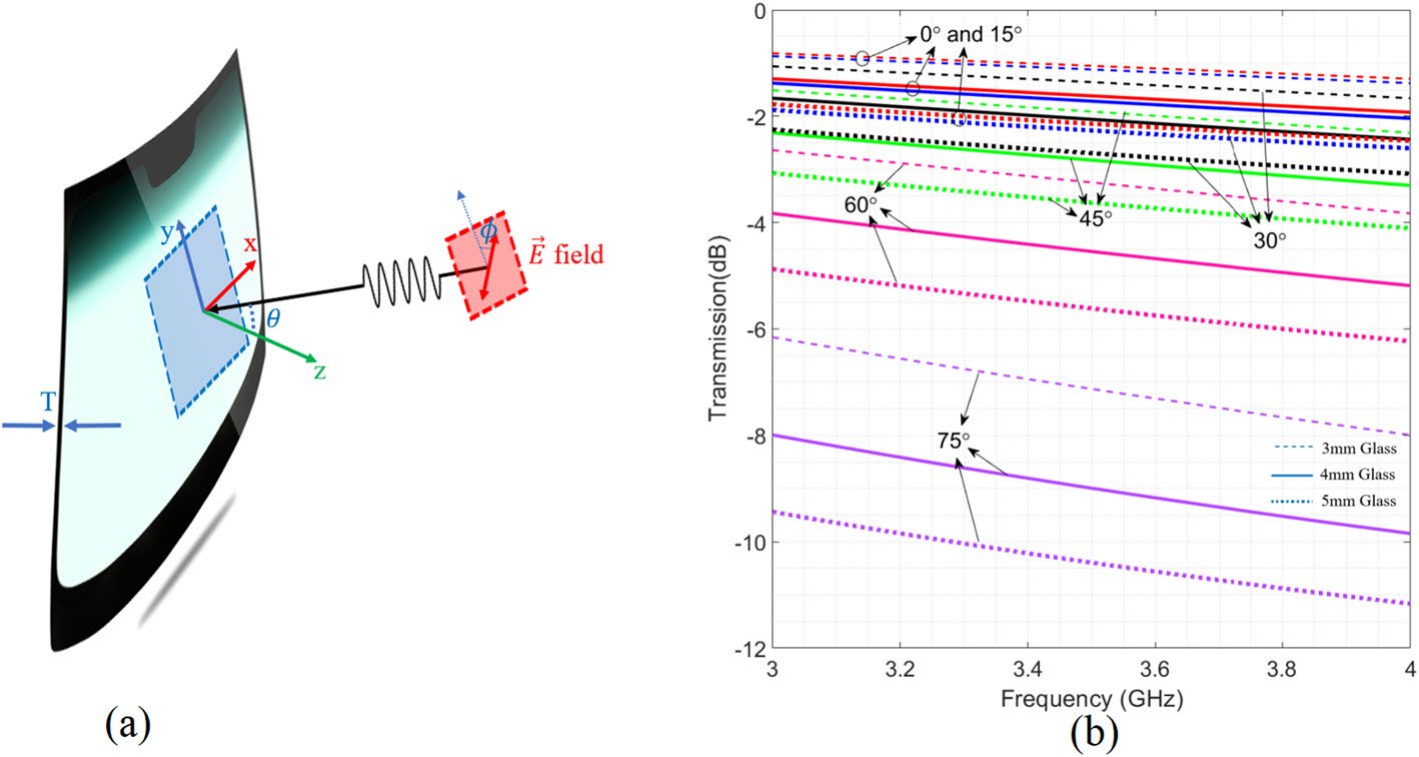
(**a**) Geometrical representation of parameters used in simulation of impinging wave on the windscreen of vehicle. (**b**) Simulation results of the transmission coefficient for single glazing glasses with different thicknesses for various $\theta$ versus frequency. All results are related to TE mode where $\phi$ is zero.

Single glazing windows are practically used in vehicles, as their windscreen or door glasses. The thickness of the windscreen is around 5 mm , and it is around 3 mm for other glasses of vehicles. Mostly, vehicular glasses are not only made from glass but still they can be modelled with permittivity values around 6^29^. Figure 1a illustrates the considered parameters and their geometrical representatives for simulating the different polarization and received angles. In this figure, a commercial sample is shown which is a curved surface. This curvature can be well-approximated with a flat surface as it varies so gently over the surface ( less than $\dfrac{\lambda }{10}$ ($\approx 8 \,\mathrm{{mm}}$) in a circular area with the radius of few wavelengths). Therefore the difference between curved and flat glasses of cars in frequency response is negligible. Beyond this criterion, some studies have used curved unit-cells providing same result with flat unit-cells owing flexible surfaces^30^. In Ref.^30^, the curvature radius is around $0.86\lambda$ and the unit-cell size is around half of the operating wavelength but still results for curved surface conform the simulation results of the flat design. In this regard, instead of curved surface, flat surface is considered for simulations and design. Figure 1b illustrates the results of transmission coefficient related to the shown structure in Fig. 1a at 3.5 GHz for TE polarization ($\phi = 0$). In this figure, TM polarization ($\phi = 90$) is not considered as it passes through the glass much better than TE mode and does not cause significant coverage problem considering the $- 3\,{\text{dB}}$ constraint for the transmission coefficient.

As shown in Fig. 1b, when the incident angle ($\theta$) is increased, the transmission coefficient drops notably which means the coverage inside the vehicle will be limited due to huge attenuation from wide incident angles. Hence we aim to address this challenge by introducing a new transparent transmission surface coated on a glass to improve the coverage inside the vehicle. It’s essential to take into account that, in vehicular applications, any modifications to the front windscreen that might affect the driver’s vision are generally avoided. As a more convenient alternative, applying the proposed metasurface to the rear windscreen is favored. The larger area available on the rear windscreen makes it an optimal location for surface installation, enhancing the chances of improving coverage.

### Propagation through double glazing glass

**Figure 2:**
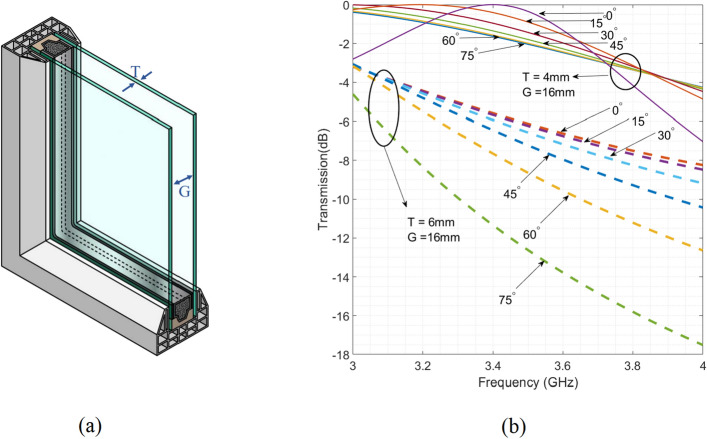
(**a**) Geometrical parameters of double glazing windows. *T* is nominally either 4 mm or 6 mm and *G* is usually 16 mm. (**b**) Transmission coefficient of the waves impinged on double glazing windows with different angles for two types of glasses with $T= 4 \,\mathrm{{mm}}$ and $T = 6\,\mathrm{{mm}}$ while the *G* is 16 mm. All results are related to TE mode where $\phi$ is zero (Fig. 1a).

Commercial glasses in the buildings are mostly double glazing due to their sound and heat transfer insulating behaviour. The geometrical parameters of a common double-glazed glass is shown in Fig. 2a, the thickness of *T* is usually 4 mm or 6 mm. The separation distance between two glasses, denoted as *G*, varies depending on the structures of the buildings. In this paper, *G* is considered 16 mm, as it provides suitable insulating behaviour and it is a common value in building construction. Therefore, in our simulation , we studied both common value of *T* (4 and 6 mm), and $G = 16\, \mathrm{{mm}}$. Therefore, their respective transmission coefficients are depicted in Fig. 2b, for various angle of incidents ($\theta$). It is illustrated in Fig. 2b that , for double glazing glass with the thickness of $T = 6\, \mathrm{{mm}}$, if $\theta$ is around $75^\circ$, the transmission power drops almost significantly by $- 12 \,\mathrm{{dB}}$ at $3.5\, \mathrm{{GHz}}$.

From Fig. 2b, it can be deduced that , for $T = 4\,\mathrm{{mm}}$, the signals around 3.5 GHz can go through the glass. However, $T = 4\,\mathrm{{mm}}$ is less favored in the building respect to the 6 mm design due to its inferior sound and heat insulation capabilities. In contrast, for $T = 6\,\mathrm{{mm}}$, as indicated in Fig. 2b, the signal transmission does not meet the $-\,3\,\mathrm{{dB}}$ constraint, even at normal incidence of $\theta =0$. As the angle of $\theta$ increases, a substantial signal attenuation is evident during the passage through windows. Therefore, our objective is to introduce a novel optically transparent transmission surface coated on double glazing glass, incorporating the specified parameters. This proposal aims to enhance coverage within buildings, addressing wide incidence angles and ensuring polarization insensitivity.

In the next section, we will present two metasurface s to address aforementioned challenges for the single and double glazing windows. The proposed transmission surfaces can be coated simply on glass surfaces, offering a versatile application. Users have the option to either attach the developed metasurface to their windows as a retrofit solution, or these surfaces can be incorporated into the manufacturing process at the outset in factories.

## Metasurface design; results and discussion

FSSs as microwave filters can be determined by their pass-band(s) and rejection-band(s), which can be dependent on the polarization of incoming waves. An FSS generally consists of one or more dielectric substrates, and a periodic arrangement of metallic unit-cells, e.g, wire grids, discreet square patches, dipole elements, etc. A dielectric substrate can be represented as an impedance mismatch by itself, and therefore when illuminated by an RF signal, partially reflects the energy. This impedance model is related to the angle of incidence and also the polarization of the wave. Varying arrangements can yield different responses like the double glazing case with $T = 4\,\mathrm{{mm}}$ and $T = 6\,\mathrm{{mm}}$. In these arrangements, considering transmission lines with respective impedances result in high or low transmission coefficients. The metallic periodic structure will engineer the reflection by tuning out the input impedance at any given operating frequency. In order to design and analyse their performance, distributed circuit models can be used to estimate the parameters of the unit-cells. This enables the manipulation of spatial filter responses for different polarisation, and incoming waves from different angles in a desired manner. In this regard, theoretical models for the unit-cells are required considering the operating frequencies, polarisation and various incoming angles. These theoretical framework are not available for all kind of FSSs, except for some simple structures. It must be noted that, single layered metallic FSSs are not capable of providing good transmission phase response^21^. Hence, designing transmission surfaces with cascaded layers can provide additional degrees of freedom (DoF), that can be exploited to improve the transmission coefficient. These kind of transmission surfaces with high transmission coefficient are a potential candidate to enhance the indoor coverage. However, cascaded layer FSSs are more sensitive to the incident angle, so developing FSS which can support wide bandwidth as well as being insensitive to wide incidence angles are challenging. Besides, the surface which is going to be coated on windows should be polarisation insensitive. This is crucial due to depolarized waves reflecting back from different scatterers in the wireless channel, where the polarization also varies over time.

A number of various designs have been investigated with extraordinary transmissions (EOT) in different frequency bands^31,32^. Although EOT structures can warranty a high transmission, their metallic connection between upper and lower surfaces renders them impractical for O2I coverage enhancement. The complexity of fabrication and the lack of optical transparency make them unsuitable for the intended purpose.

**Figure 3:**
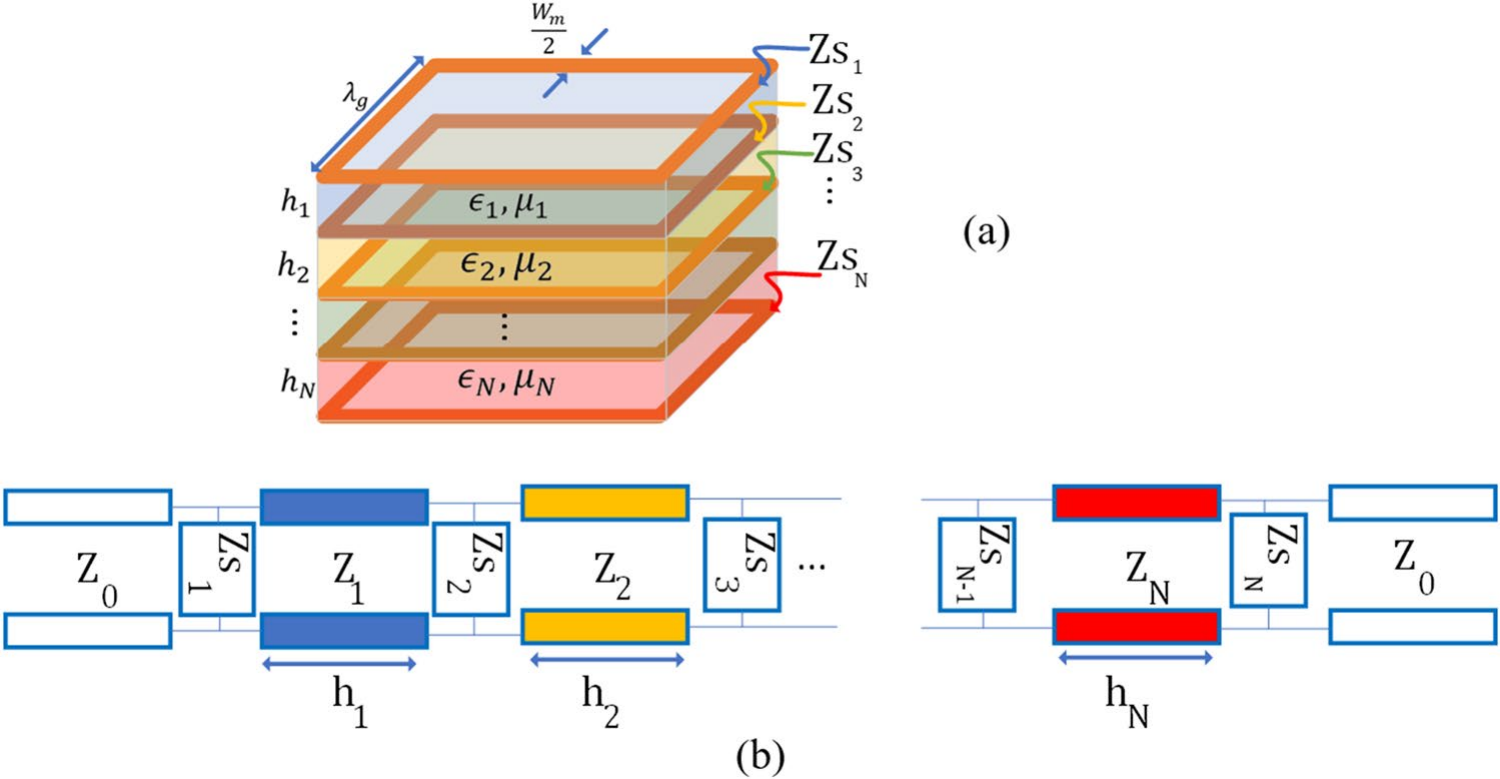
General structure of layered medium (**a**) Stacked layers of metasurfaces and materials with different permittivity and permeability. In this shape, grids (square ring patch) are assumed to provide different surface impedances. (**b**) Circuit model for stacked layers.

In this paper, we introduce a novel technique to achieve extraordinary transmission that maintains transparency, and notably, there is no connection between the upper and lower surfaces. This design makes fabrication significantly easier and more practical. The theory behind of the proposed technique is based on the boundary condition for PEC surfaces. Basically, when the thickness of dielectric substrates between conductive screen is much less than the wavelength of operating frequency, the field distribution remains almost identical to that of a scenario where there is a metallic connection between upper and lower surfaces. Therefore, similar results are expected to be achieved.

To synthesise any particular EOT structures, different circuit models are proposed^33,34^ which they can be also considered for modelling cascaded FSSs, by defining a suitable surface impedance. Based on the geometrical parameters of the simple grid, which are shown in Fig. 3a, the elements in the circuit model (Fig. 3b) can be expressed as below^33,34^:

1 $$\begin{aligned} Z_g = \dfrac{j\omega \eta _0 \lambda _g}{2\pi c} \ln \bigg (\dfrac{1}{\sin \big (\dfrac{\pi w_m}{2 \lambda _g}\big )}\bigg ), \end{aligned}$$

where the $\eta _0$ and *c* represent the free-space impedance and speed of light , respectively. Additionally, other parameters are as below:

2 $$\begin{aligned} Z_0 = \eta _0, Z_d^n = \dfrac{\eta _0\sqrt{\mu _r^n}}{\sqrt{\epsilon _r^n (1-j \tan (\delta ))}}, \end{aligned}$$

where $\epsilon _r^n$ and $\mu _r^n$ represent the relative permittivity and permeability of $n{\mathrm{{th}}}$ layer, respectively. These equations have been developed for grids under normal incident angles^34^. For tilt angles, there are approximate analytical^35^ and numerical models^33^ available. It’s worth mentioning that these equations are specifically applicable to normal grids, restricting their applicability to other grid configurations.

These models are considering the PEC boundary condition for the conducting surfaces of the grids. However, in our design, we use Indium Tin Oxide (ITO) to achieve an optically transparent metasurface. It is noted that, ITO has limited conductivity in microwave frequencies, significantly lower than materials like copper. The induced currents then will propagate through the material instead of being limited to the surface of conducting material due to the limited conductivity. Induced currents in the conductive sheets can be assumed confined in a region with the thickness of $\sqrt{\dfrac{2}{\omega \mu _0\sigma }}$ beneath the surface of conductive material which is called skin depth. If the conducting layer’s thickness is higher than the skin depth, this model is applicable^36^ and can be utilised for modeling the structure shown in Fig. 3a. In practical cases, as the thickness of conductive sheet is much less than the relevant skin depths, conductive layers are coupled to each other and Eqs. (1) and (2), wouldn’t be valid anymore. Therefore, full-wave simulations for FSSs with EOT performance is needed^34^. However, Eqs. (1) and (2), can be used for devising initial points for optimizing the design in full-wave simulator.

### Transmission surface for extending the coverage inside vehicles

The circuit model presented in Fig. 3b, can be used for both single glazing and double glazing glasses. As mentioned earlier, for the vehicular glasses, the thickness of side windows is 3 mm, while for the rear and front windscreens is 5 mm. TE polarized incoming waves experience huge attenuation compared to TM modes when propagating through the glass, particularly at higher angles of incidence (see Fig. 1b). Here, we consider this model with a grid metallic pattern to design a transmission surface based on single-glazed glass to enhance the transmission bandwidth. However, this model can not directly be used for polarisation and maximizing the angle stability of the surface since the surface impedance of the structure is dependent to the incident angle. It worth mentioning that deriving equations for other incident angles based on Eqs. (1) and (2) is not thoughtful, as these equations represent the impedance in lossless scenarios. In practical applications, grids are often lossy and consequently coupled to each other, introducing additional complexities that these equations do not account for. This coupling also increases in thin solutions while they are more favored in practical applications. Thin solutions are results of stacking few layers of metallic structures and dielectric substrates. Additionally, for more complex metalic structures, there is no model to be used in theory and their impedance behaviour should be derived numerically. As a result, there is no theoretical framework to develop a polarisation insensitive metausrface with high transmission over a wide incident angles. It must be noted that, unit-cell metallic shape, number of stacked layers (*N*), geometrical parameters of the grid ($\lambda _g, w_m$), the permittivity and the thickness of layers are key parameters of the design. In our proposed structures, the thickness and permittivity of the glass layer(s) are imposed from the current commercial glasses and are fixed. Based on the limitation dictated by commercial glasses available in the market, optimization parameters are limited to devise *N*, $\lambda _g$ and $w_m$ for the grid unit-cell shown in Fig. 3a and also the order of arranging these layers.

**Figure 4:**
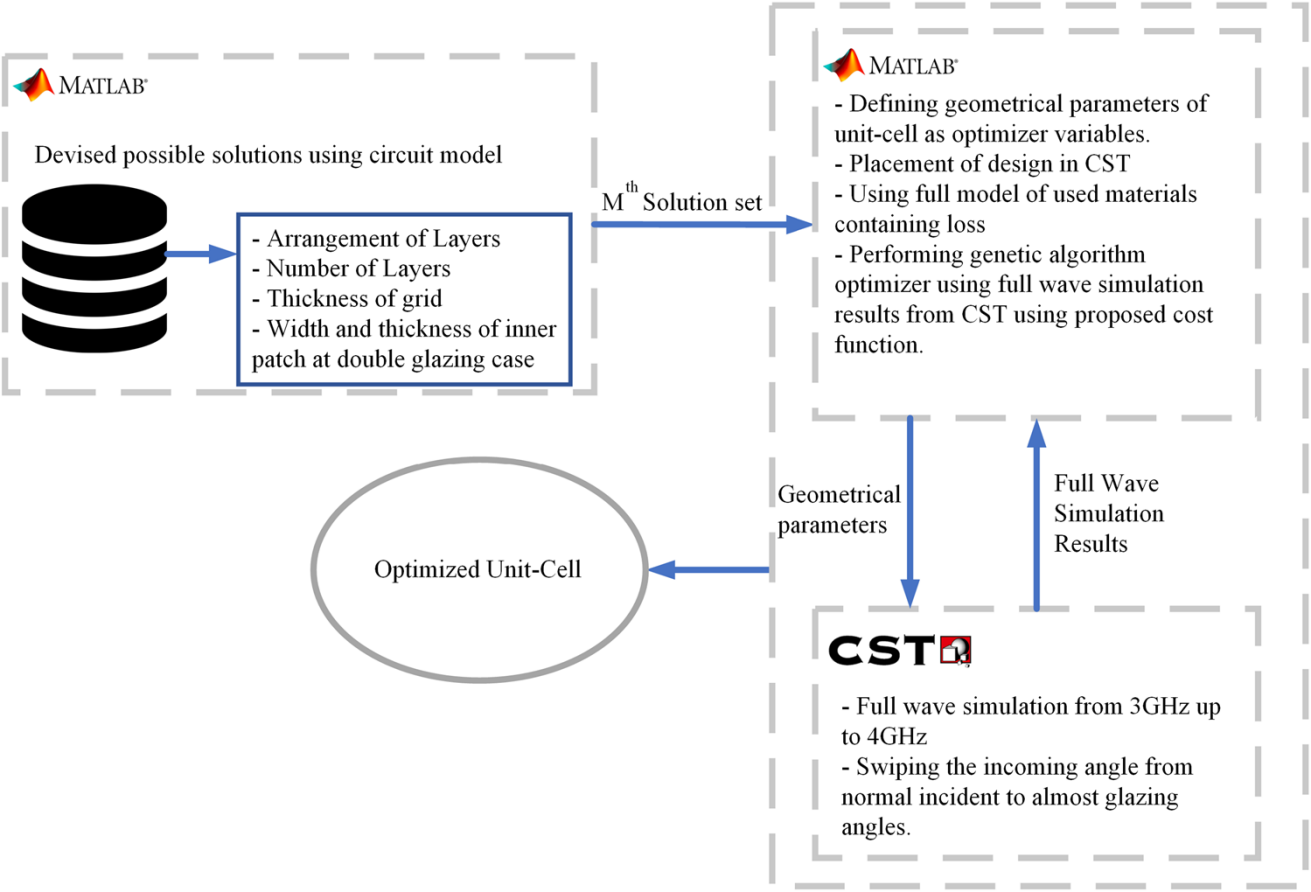
Actor-critic structure for maximizing the the acceptable maximum angle of incidence with acceptable transmission coefficient all over the *n*77 and *n*78 bands.

As a first step, Eqs. (1) and (2) have been utilised to initiate several solutions with high transmission in *n*77 and *n*78 frequency bands when only normal incidence angle is considered. Therefore, all of these designs require further optimisation to enable them to support propagation through glasses for wide incident angles. In the proposed structure, the conductive screen is composed of ITO, which, while being a relatively good conductor in microwave frequencies, experiences Ohmic loss in practical applications. (The resistivity of the used sheet is $1\,\Omega /{\text{m}}^2$). Hence, in our analysis, we need to consider the skin depth, as the induced currents are not limited to the surface of conductive screen. Then the structure gets more complex, as skin depth is higher than the thickness of conducting layers in the proposed metasurface. Therefore, induced currents in different layers are coupled to each other and full-wave simulations are required to completely characterize the response of the structure.The full-wave simulation, not only considers the skin-depth but also in our designing procedure is used to reduce the the sensitivity to incident angle while maintaining high transmission coefficient. However, relying on try and error approaches to develop a new surface is time consuming. Here, to devise the unit-cell’s parameter, evolutionary optimizing algorithms e.g, genetic algorithm is used. Figure 4, expresses the diagram of our simulation procedure for designing suitable unit-cell in which the genetic algorithm as an optimizer tool. As shown in Fig. 4, in our algorithm, the predefined genetic algorithm in MATLAB is used to optimize the design in CST. In our proposed designing procedure, initial possible candidates are used to improve the speed of convergence to optimal solution. Possible candidates are achieved using the previously shown circuit model in Fig. 3. In this regard, configuration sets containing parameters like the number of stacked layers, for all possible solutions capable of providing high transmission coefficient are stored. These sets are then used in the algorithm as starting points for the optimization process. Therefore, all sets of the *N*, $\lambda _g$ and $W_m$ which are capable of providing high transmission coefficient at 3.5 GHz for the normal incident angle are considered. In this stage, various permutations of stacked layers are also considered. The optimized cost function in this algorithm is devised to guarantee the high transmission coefficient from 3.4 up to 3.6 GHz,and, at the same time, to assist the optimizer in converging to a design capable of maintaining high transmission from zero up to higher angles of incidence for both TE and TM polarizations.

The below list of bullet points, expresses the proposed pseudo-code which is used to in our algorithm to assign score to any design and has been used in optimization process in Fig. 4. For the single glazing scenario, among the designs having same score, the one proposing higher frequency bandwidth is selected as optimum solution.
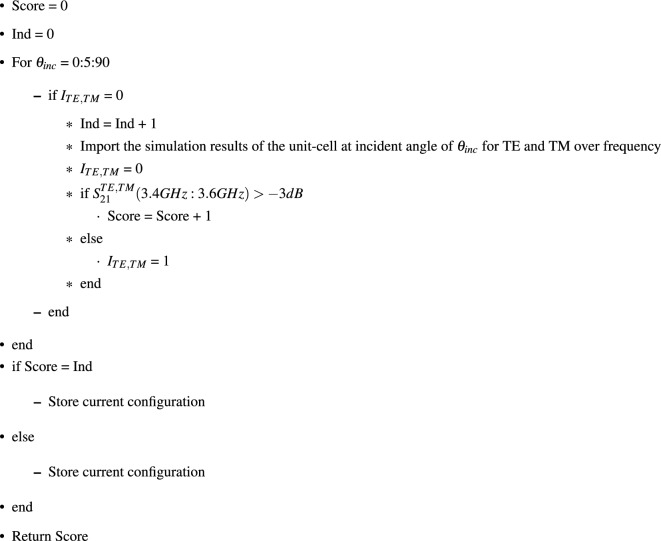


**Figure 5:**
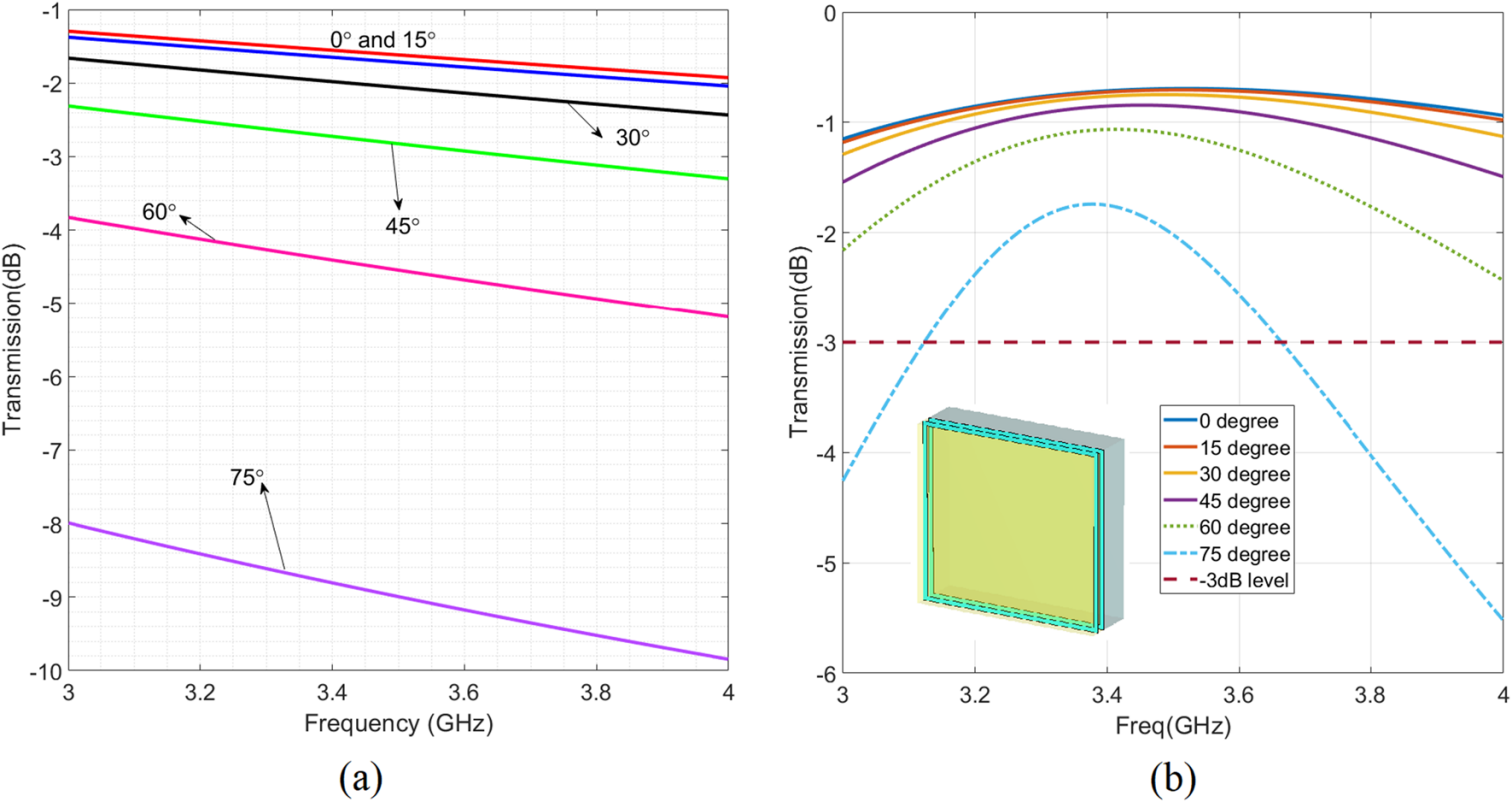
Simulation results of (**a**) Normal glass with the thickness of 4 mm for TE polarization. (**b**) Proposed unit-cell for single glazing design which shows 600 MHz bandwidth with higher than $-3\,\mathrm{{dB}}$ for transmission coefficient up to 75 degrees.

Figure 5b represents the shape of unit-cell for single-glazed glass along with the respective transmission coefficient as a function of frequency for different angles of incidence. From the optimisation tool, unit-cell with two conductive layers has been acquired. To maintain the transparency of the whole structure BOROFLOAT33 glass is considered as a substrate with the permittivity of 4.5 and thickness of 1.1 mm. Its loss tangent is also around 0.006. The conductive screen is ITO which is optically transparent good conductor in microwave frequencies. The resistance of the ITO is around $1\, \Omega /\mathrm{{m}}^2$. Proposed metasurface is coated on glass with the order of BOROFLOAT/ITO/BOROFLOAT/ITO. As it can be seen from Fig. 5a,b, the proposed metasurface improve s the signal strength through a single-glazed glass for incidence angles between $0^\circ$ to $75^\circ$ over 600 MHz bandwidth.

**Figure 6:**
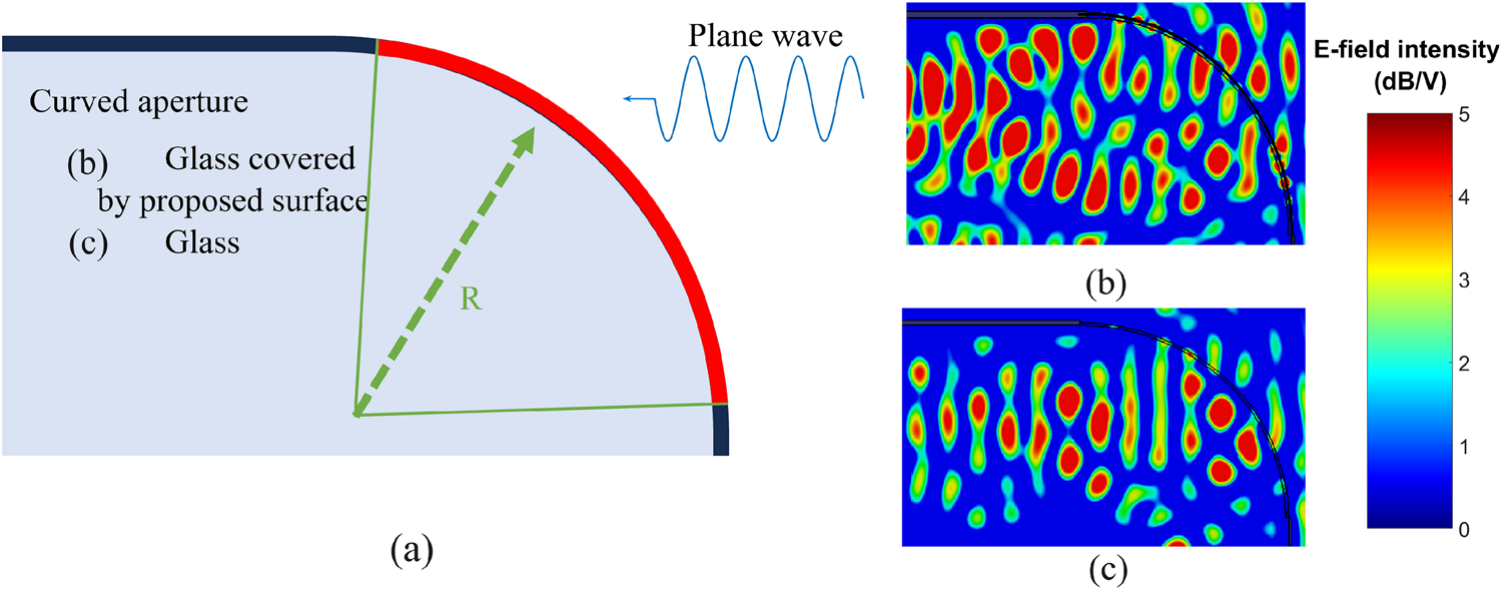
Proposed setup for taking curved structures into consideration. (**a**) Proposed structure for curved surfaces. This structure takes into account a superposition of all angles impinged on the curved surface with *R* as its radius. (**b,c**) Express the simulation results of the curved glass with $R = 0.5\,\mathrm{{m}}$ for both with and without our proposed design, respectively. Both figures use same scales.

Additionally, as shown in Fig. 6a, a curved aperture is used in simulation to investigate the effect of curved surfaces and response of our proposed design in the case of being used in conformal surfaces. This curved surface has the radius of 0.5 m and different segments of this design are receiving signals at different angles as the signal is coming from right side of Fig. 6a toward left. Incident angles at different segments of explained structure, varies from 0 to 90 degrees locally. Figure 6b depicts the simulation result of the curved glass with metasurface while the shown figure in Fig. 6c, expresses the result of the normal glass (without metasurface). By considering the shown field intensity in proximity to the glass inside the room (light blue section in Fig.6a) at both depicted results, the glass with metasurface provides higher field intensities specifically at higher parts of the glass, where the angle of incident is higher.

using our proposed structure on a curved glassy window with the radius of 0.5 m is improving the indoor coverage. In this figure, a plane wave is impinging on the curved surface from right to left. Simulation results belong to the 3.5 GHz.

### Transmission surface for enhancing the coverage inside buildings

**Figure 7:**
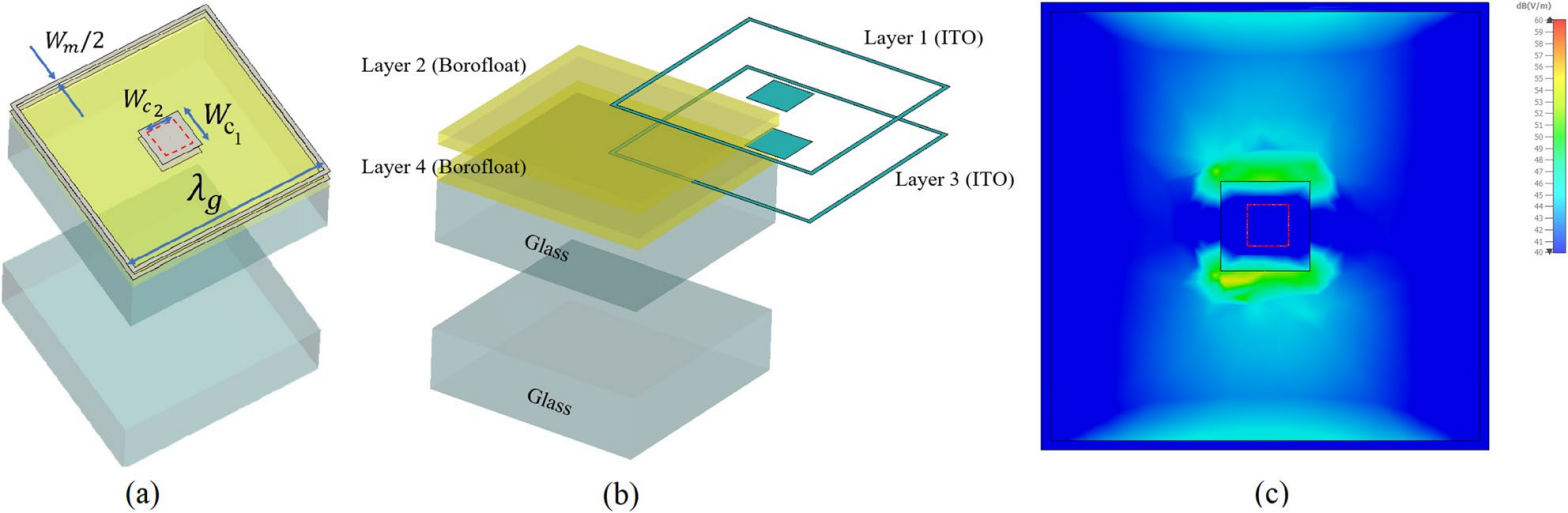
Proposed unit-cell for double glazing solution (**a**) Geometrical representation. (**b**) Stacking order of double glazing proposed solution. (**c**) Field distribution of the unit-cell. Dashed red square border line represents the suitable region for extraction to decrease the opacity.

Modern building architectures do not consider the necessity for radio signals to go through buildings. For instance, numerous modern office buildings are constructed with metal cladding and low-emission double-glazed glass, which severely hinders propagation into buildings. Hence, to enhance in-building propagation, a new metasurface which is transparent, insensitive to the polarisation, with high transmission coefficient over wide range of incident angles is required, which is proposed in this section. Similar to previous section, an actor-critic approach, along with the circuit model shown in Fig. 3b, is employed to optimise the parameters of the unit-cell. Firstly, all possible initial solutions of normal incidence with high transmission coefficient for the proposed structure related to double-glazed glass are predicted using the same design in Fig. 3a. However, using the same structure in Fig. 3a, proves inadequate in ensuring high angle stability, even though it can deliver a high transmission coefficient under normal incidence. Therefore, the unit-cell design requires further amend in the shape and also possibly in the placement order of stacked layers. The double-glazed window is constructed from two panes of glass with the thickness of 6 mm, which are separated by a 16 mm air gap. In contrast with single-glazed unit-cell, the general representation of optimum unit-cell for double-glazed is supposed to have an inner patch, shown in Fig. 7a, to improve the angle stability and frequency response. For further improvement of the optical transparency and reducing the ohmic loss of the proposed structure, the inner patch is replaced by a ring patch. Fig. 7b represents the further details of the proposed unit-cell in Fig. 7a, including the stacking order of layers. Figure 7c represents the field distribution over the surface of the proposed unit-cell and it can be seen that by extracting the area delineated by the red boundaries, not only is the field distribution unchanged, but also the optical transparency can been improved. Therefore, the inner parts of the square-shaped patch can be extracted since the field intensity in these areas is considerably lower than in the outer regions. Extracting the middle part of the patch does not significantly impact the overall frequency response of the design. Among the shown parameters in Fig. 7a, the ${W_c}_{2}$, represents the dimension of this region and other parameters like ${W_c}_{1}$, $\lambda _g$, and $\dfrac{W_m}{2}$ express the size of patch, unit-cell and width of the grid, respectively, and have been achieved from described optimization process. In this optimization process, using better initial points for the design not only enhanced the convergence speed toward the optimum solution but also facilitated the determination of parameters for the optimal solution. This automated approach proved invaluable in identifying parameters that might be overlooked in a blind search.

**Figure 8:**
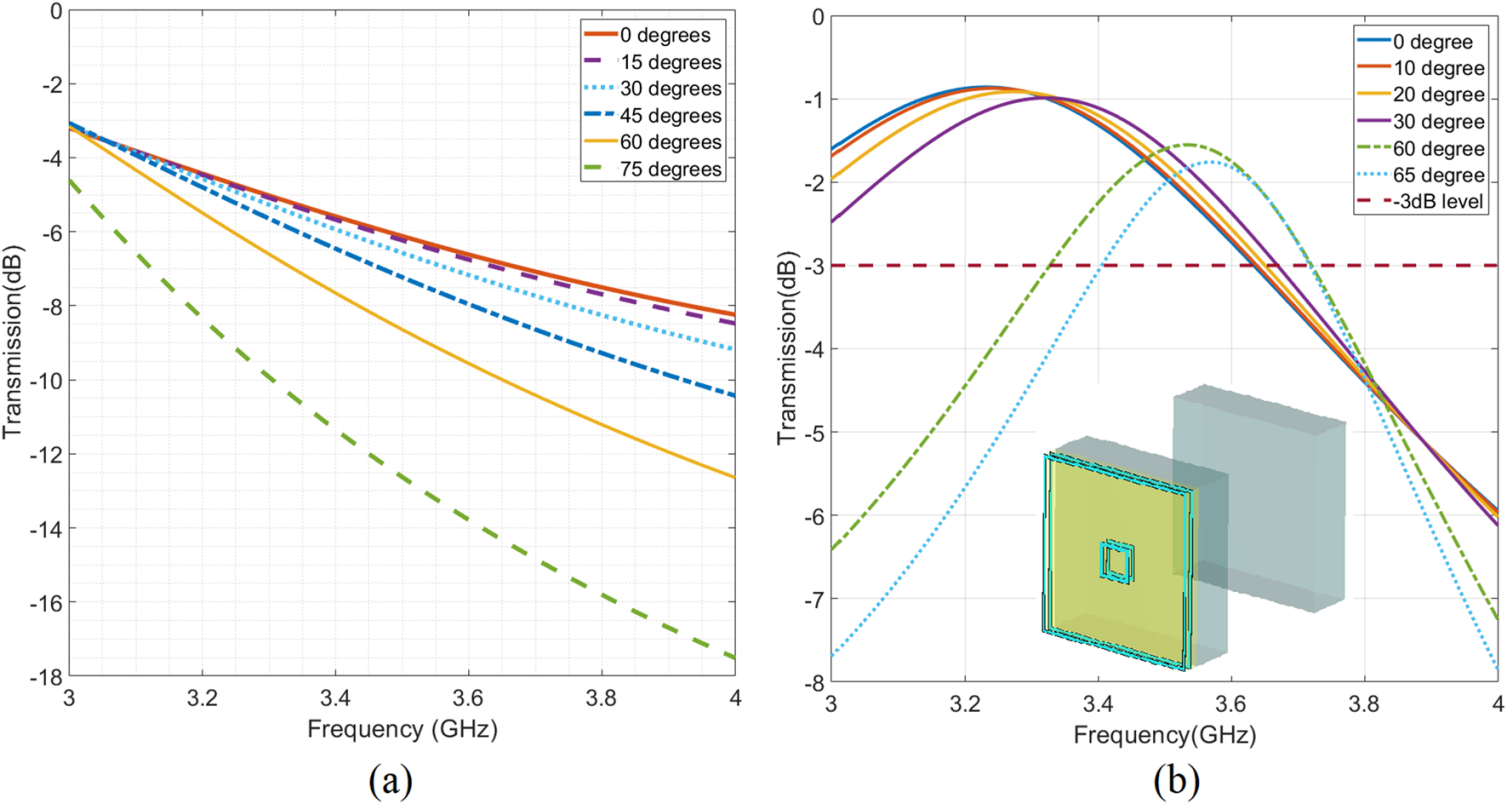
Simulation results of (**a**) Normal double glazing glass ($T = 6\,\mathrm{{mm}}$ and $g = 16\, \mathrm{{mm}}$) for TE polarization. (**b**) Proposed unit-cell for double Glazing design which shows 200 MHz bandwidth with higher than $-\,3\,\mathrm{{dB}}$ for transmission coefficient up to 65 degrees.

In both of single- and double-glaze d solutions, by using back and forth procedure as explained in Fig. 4, between MATLAB and CST, while scoring different designs, led to the derivation of the final design capable of providing highest angular stability (Fig. 4). The simulation results for the final unit-cell of double glazing windows for different incident angles are presented in Fig. 8b while Fig. 8a represents the related results to bare double-glazed glass. The final unit-cell is double-layered and including two patch ring that will be coated in a periodic manner on just outer side of one of the the float glasses with the order of ITO/BOROFLOAT/ITO/BOROFLOAT. In this case, the construction and installation of such a metasurface are easy since there is no need to coat both glasses with the proposed metasrurface. As it is shown in Fig. 8b, the proposed metasurfce is capable of providing a high transmission coefficient up to $65^\circ$ over almost 200 MHz, meeting the $- 3\,\mathrm{{dB}}$ threshold requirement, which is sufficient for 5G application. Additionally, it shows the strength of the proposed method as it can be seen that while it may not yield an exceptionally high transmission coefficient for normal incidence, it excels in providing high angular stability.

Geometrical parameters of the proposed unit-cells can be expressed by $\lambda _g = 20\,\mathrm{{mm}}$ for both of the unit-cells while $w_m$ is $0.5\,\mathrm{{mm}}$ and $0.8\,\mathrm{{mm}}$ for single-glazed and double glazing windows, respectively. $W_{c1}$ and $W_{c2}$ are 4 mm and 3 mm, respectively. Therefore, the dimension of unit-cells is less than $\lambda /4$, and the thickness of used BOROFLOAT glasses are 1.1 mm.

### Measured results

The issue of indoor coverage caused by single and double glazing glasses is shown in Fig. 10a,b. It is worth mentioning that two antennas are placed on two sides of the glasses and the effect of different glasses is measured and depicted in Fig. 10a,b. It can be seen from Fig. 10a,b that, even at normal angle of incidence, the transmission coefficient is not particularly high, especially in the case of double glazing scenario. Therefore, indoor scenarios suffer from coverage issues even at non energy efficient cases which are the cases that glasses are covered by conductive transparent sheets to block the heat transfer between outdoor and indoor.

**Figure 9:**
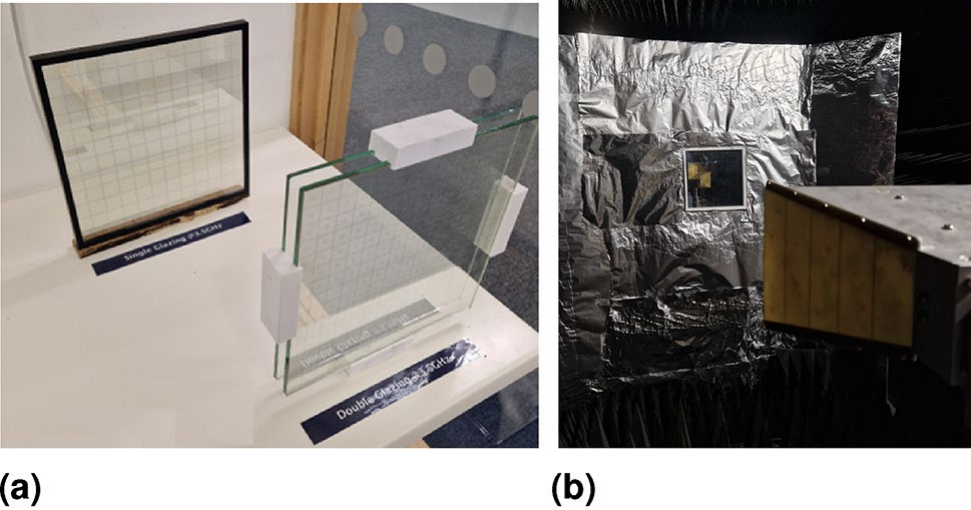
Fabricated designs (Left) and their measurement setup in chamber room (Right).

**Figure 10:**
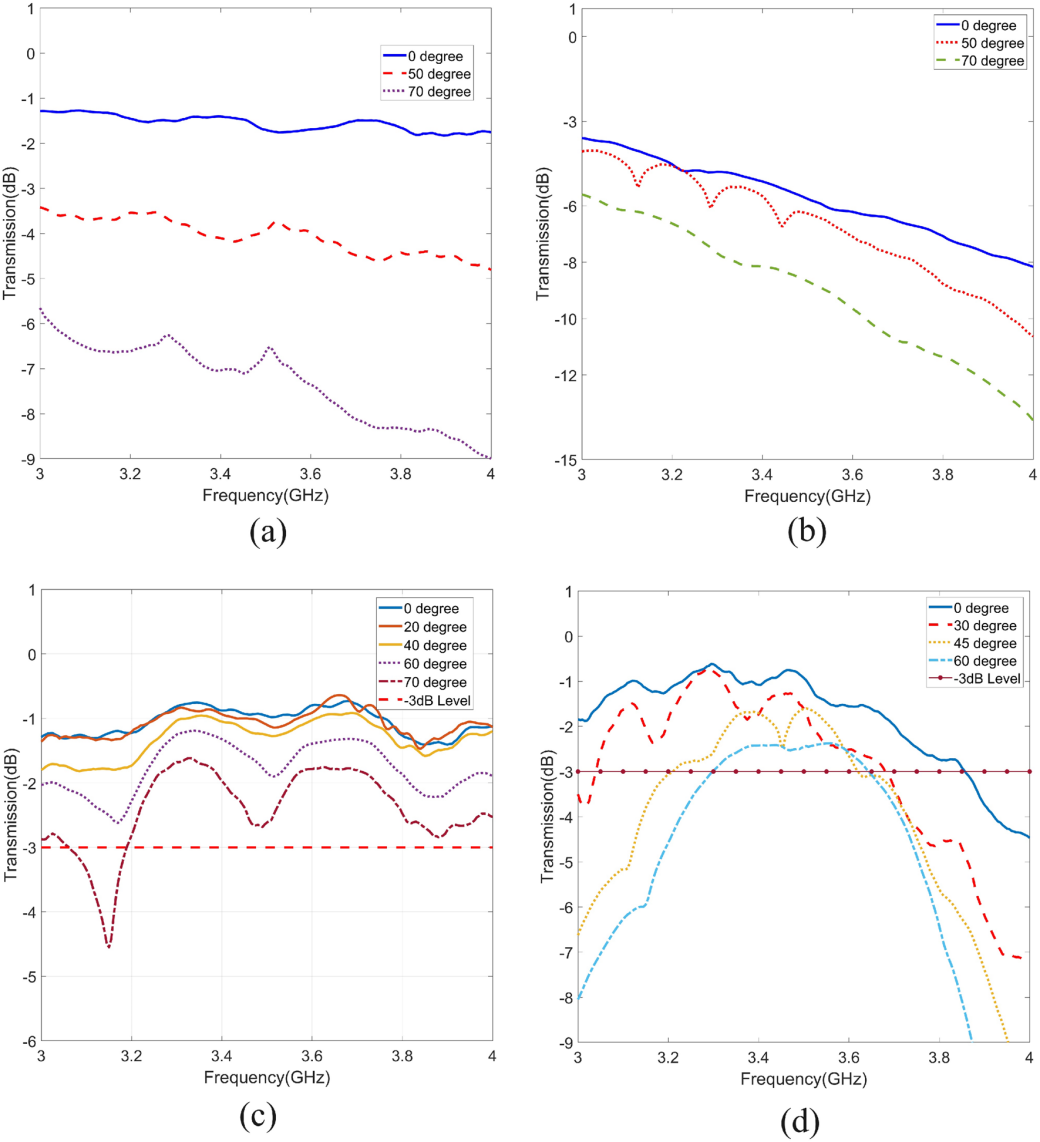
Measurement results of the transmission coefficients for different scenarios of glasses. These sub-figures belong to single and double glazing cases. (**a**) Single glazing window without metasurface. As shown in this sub-figure, at high angles of incidence, the transmission coefficient is low and not suitable according to noted criteria (> − 3 dB). (**b**) Transmission coefficient for a normal double-glazed glass surfaces without metasurface is shown. Even at normal incident, wave transmission is not high. (**c,d**) Represent the transmission coefficient after attaching proposed metasurface to the single and double glazing glasses respectively.

**Figure 11:**
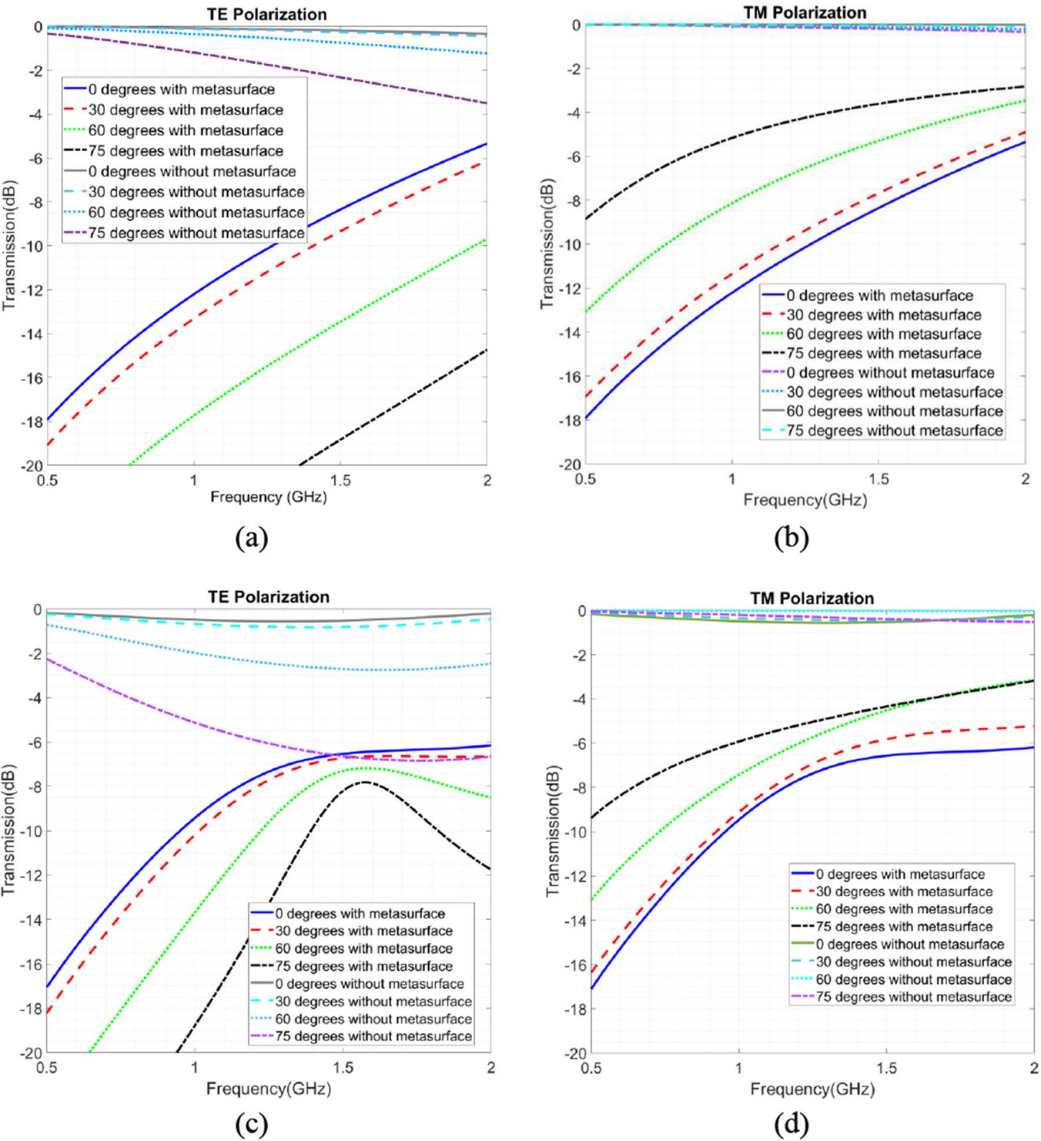
Simulation results of the conventional glasses at lower frequencies related to previous mobile generations for both cases of with and without designed metasurfaces. (**a,b**) Represent TE and TM polarization responses for single glazing solution and (**c,d**) express related results to TE and TM polarization for double glazing solution.

Both metasurfaces have been fabricated and verified experimentally. Figure 9 depicts the fabricated designs and the measurement setup in the chamber room. In this setup two high gain horn antennas are used to impinge on the surface of the designed structures. The structure is desired to simulate the behaviour of typical building walls, employing stacked layers of aluminum foils. The dimensions of the setup are carefully chosen to ensure that when the aperture is shielded with a metallic plate, the received signal at the receiver remains at approximately the noise level. This characteristic enables the structure to prevent signal penetration, allowing the measurement setup to independently account for signal penetration through the glass. As mentioned earlier, in both of the designs, designed layers can be simply attached to the commercial glasses without blocking the vision of user. Both of the metasurfaces are transmitting more than 80% of the light. Fabricated surfaces are 30 cm by 30 cm and the aperture inside the wall is slightly bigger than glasses in order to fit the surfaces with their frames. The wall is equipped with two pairs of hinges, facilitating easy adjustment of the surfaces to various angles in front of the antennas. The height of the noted wall is around 2 m, with the width of the central segment being around 1 m and the side walls having widths of 0.5 m each. Middle section of the wall has an aperture with the dimension of around $33\, \mathrm{{cm}} \times 33\, \mathrm{{cm}}$, designed to accommodate the glasses along with their frames. Ideally, the frames of the fabricated glasses should perfectly fit the aperture; however, in practical scenarios, any gaps between the frames and the aperture should be filled with metals or high-quality absorbers. This consideration becomes particularly crucial in double glazing scenarios, where, at high angles of incidence, the air gap within the double-glazed setup demands careful attention for optimal performance. Measurement results (Fig. 10) represent that the transmission remains higher than $- 3 \,\mathrm{{dB}}$ up to almost 70 degrees in the single-glaze design from almost 3.2 GHz to 4 GHz. Same measurement for the double glaze solution represents that it can achieve a high transmission coefficient up to approximately 60 degrees, covering almost 200 MHz around 3.5 Hz.

The evaluation of our proposed designs for single and double glazing solutions should take into account their responses in comparison to previous generations of mobile communication. In Fig. 11 we illustrate the frequency responses of conventional glasses alongside those of our designed structures across a frequency range of 0.5 Hz to 2 GHz. This analysis considers various angles of incidence for both TE and TM polarizations. As expressed in Fig. 11, our proposed structure exhibits a minimal impact on signal attenuation when compared to conventional glasses without metasurfaces. Notably, the signal attenuation is not significantly pronounced, suggesting the effectiveness of our solution. Additionally, since coverage issues tend to be less prominent in lower frequencies, our proposed solution demonstrates practical applicability in real-world scenarios.

## Conclusion

In this paper, we have presented two transmissive metasurfaces that can be simply coated on glass surfaces to improve their frequency response in terms of signal transmission for incoming signals with different polarization over a wide range of incident angles. Two different symmetrical unit-cells have been designed through optimisation technique to enhance the transmission over wide incidence angles. As a result, high transmission up to 75 degrees for the single glazing case and up to 65 degrees for the double-glazed glasses have been obtained. The fabricated transmission surfaces are capable of enhancing 5G signal propagation through vehicles and buildings at *n*77 and *n*78 frequency bands. Besides, they are optically transparent and easy to install. Maximum propagation enhancements of 12 B and 8 B have been demonstrated for double- and single-glazed glass, respectively. If propagation losses to the most inaccessible parts of the office buildings could be reduced by around 3 B using the proposed surfaces, there is a potential to double spectrum efficiency in *n*77 and *n*78 bands through increased frequency re-use. The fabricated structures are evaluated experimentally, and their results proves the suitable performance of both metasurfaces, making them a promising solution for O2I coverage enhancement. Based on the measured results, it is recommended that future buildings take into consideration radio propagation during the design phase. Additionally, this study can be further improved by considering previous generations of mobile communication using dual(multi) band metasurfaces.

## Data Availability

The data that support the findings of this study are available from the corresponding author upon reasonable request.

## References

[1] Wang Y, Feng G, Sun Y, Qin S, Liang Y-C (2020). Decentralized learning based indoor interference mitigation for 5g-and-beyond systems. IEEE Trans. Veh. Technol..

[2] Shakhatreh H, Khreishah A, Khalil I (2018). Indoor mobile coverage problem using UAVS. IEEE Syst. J..

[3] Rudd, R., Craig, K., Ganley, M. & Hartless, R. Building materials and propagation. *Final Report, Ofcom*, Vol. 2604 (2014).

[4] Araghi A (2022). Reconfigurable intelligent surface (RIS) in the sub-6 GHz band: Design, implementation, and real-world demonstration. IEEE Access.

[5] Wu T (1995). Frequency Selective Surface and Grid Array.

[6] Munk BA (2000). Frequency Selective Surfaces: Theory and Design.

[7] Li B, Shen Z (2013). Three-dimensional bandpass frequency-selective structures with multiple transmission zeros. IEEE Trans. Microw. Theory Tech..

[8] Lee J, Lee B (2016). Design of thin RC absorbers using a silver nanowire resistive screen. J. Electromagn. Eng. Sci..

[9] Hussain N, Kedze KE, Park I (2017). Performance of a planar leaky-wave slit antenna for different values of substrate thickness. J. Electromagn. Eng. Sci..

[10] Liu N, Sheng X, Zhang C, Guo D (2017). Design of frequency selective surface structure with high angular stability for radome application. IEEE Antennas Wirel. Propag. Lett..

[11] Winkler SA, Hong W, Bozzi M, Wu K (2010). Polarization rotating frequency selective surface based on substrate integrated waveguide technology. IEEE Trans. Antennas Propag..

[12] Syed IS, Ranga Y, Matekovits L, Esselle KP, Hay SG (2014). A single-layer frequency-selective surface for ultrawideband electromagnetic shielding. IEEE Trans. Electromagn. Compat..

[13] Li D, Li T-W, Li E-P, Zhang Y-J (2017). A 2.5-d angularly stable frequency selective surface using via-based structure for 5G emi shielding. IEEE Trans. Electromagn. Compat..

[14] Sarabandi K, Behdad N (2007). A frequency selective surface with miniaturized elements. IEEE Trans. Antennas Propag..

[15] Ghosh S, Lim S (2018). Fluidically reconfigurable multifunctional frequency-selective surface with miniaturization characteristic. IEEE Trans. Microw. Theory Tech..

[16] Danesh, S., Bagheri, A. & Khalily, M. Wide-incidence angle and polarisation insensitive transparent metasurface for 5g outdoor to indoor coverage enhancement. In *2022 IEEE International Symposium on Antennas and Propagation and USNC-URSI Radio Science Meeting (AP-S/URSI)* 239–240 (IEEE, 2022).

[17] Chen H, Chen H, Xiu X, Xue Q, Che W (2021). Transparent FSS on glass window for signal selection of 5g millimeter-wave communication. IEEE Antennas Wirel. Propag. Lett..

[18] Sharma SK, Zhou D, Luttgen A, Sarris CD (2018). A micro copper mesh-based optically transparent triple-band frequency selective surface. IEEE Antennas Wirel. Propag. Lett..

[19] Li H, Wang G, Cai T, Hou H, Guo W (2019). Wideband transparent beam-forming metadevice with amplitude-and phase-controlled metasurface. Phys. Rev. Appl..

[20] Jia SL, Wan X, Su P, Zhao YJ, Cui TJ (2016). Broadband metasurface for independent control of reflected amplitude and phase. AIP Adv..

[21] Yang F, Deng R, Xu S, Li M (2018). Design and experiment of a near-zero-thickness high-gain transmit-reflect-array antenna using anisotropic metasurface. IEEE Trans. Antennas Propag..

[22] Abdelrahman AH, Elsherbeni AZ, Yang F (2013). Transmission phase limit of multilayer frequency-selective surfaces for transmit array designs. IEEE Trans. Antennas Propag..

[23] Anwar RS, Mao L, Ning H (2018). Frequency selective surfaces: A review. Appl. Sci..

[24] Yang F, Rahmat-Samii Y (2019). Surface Electromagnetics: With Applications in Antenna, Microwave, and Optical Engineering.

[25] Farooq U, Shafique MF, Mughal MJ (2019). Polarization insensitive dual band frequency selective surface for RF shielding through glass windows. IEEE Trans. Electromagn. Compat..

[26] Chatterjee A, Parui SK (2020). A triple-layer dual-bandpass frequency selective surface of third order response with equivalent circuit analysis. Int. J. RF Microwave Comput. Aided Eng..

[27] Lin B-Q, Li F, Zheng Q-R, Zen Y-S (2009). Design and simulation of a miniature thick-screen frequency selective surface radome. IEEE Antennas Wirel. Propag. Lett..

[28] Xu N, Gao J, Zhao J, Feng X (2015). A novel wideband, low-profile and second-order miniaturized band-pass frequency selective surfaces. AIP Adv..

[29] Ruddle, A., Zhang, H., Low, L., Rigelsford, J. & Langley, R. Numerical investigation of the impact of dielectric components on electromagnetic field distributions in the passenger compartment of a vehicle. In *2009 20th International Zurich Symposium on Electromagnetic Compatibility* 213–216 (IEEE, 2009).

[30] Liang B, Sanz-Izquierdo B, Parker EA, Batchelor JC (2014). Cylindrical slot FSS configuration for beam-switching applications. IEEE Trans. Antennas Propag..

[31] Aközbek N (2012). Experimental demonstration of plasmonic Brewster angle extraordinary transmission through extreme subwavelength slit arrays in the microwave. Phys. Rev. B.

[32] Bagheri A, Rahmani B, Khavasi A (2017). Effect of graphene on the absorption and extraordinary transmission of light in 1-d metallic gratings. IEEE J. Quantum Electron..

[33] Molero C, Rodriguez-Berral R, Mesa F, Medina F (2017). Wideband analytical equivalent circuit for coupled asymmetrical nonaligned slit arrays. Phys. Rev. E.

[34] Kaipa CS (2010). Circuit modeling of the transmissivity of stacked two-dimensional metallic meshes. Opt. Express.

[35] Padooru YR, Yakovlev AB, Kaipa CS, Medina F, Mesa F (2011). Circuit modeling of multiband high-impedance surface absorbers in the microwave regime. Phys. Rev. B.

[36] Kiani, G., Olsson, L., Karlsson, A. & Esselle, K. Transmission analysis of energy saving glass windows for the purpose of providing FSS solutions at microwave frequencies. In *2008 IEEE Antennas and Propagation Society International Symposium* 1–4 (IEEE, 2008).

